# Orientation tuning of a two-stimulus afterimage: Implications for theories of filling-in.

**DOI:** 10.2478/v10053-008-0002-7

**Published:** 2008-02-15

**Authors:** Daniel R. Van Horn, Gregory Francis

**Affiliations:** 1 Psychological Sciences, Purdue University,West Lafayette, IN, USA; 2 Laboratory of Psychophysics, Brain Mind Institute, École Polytechnique Fédérale de Lausanne, Switzerland

**Keywords:** afterimage, brightness perception, filling-in

## Abstract

Sequential viewing of 2 orthogonally related gratings produces an afterimage related to the firstgrating (Vidyasagar, Buzas, Kisyarday, & Eysel, 1999; Francis & Rothmayer, 2003). We investigated how the appearance of the afterimage depended on the relative orientations of the 2 stimulus gratings. We firstanalyzethetheoretical explanation of the appearance of the afterimage that was proposed by Francis and Rothameyer (2003). From the analysis, we show that the model must predict a rapid drop in afterimage occurrence as the gratings deviate from orthogonal. We also show that the model predicts that the shape of the afterimage should always be orthogonal to the second grating. We then report on 2 experiments that test the properties of the model and find that the experimental data are strikingly different from the model predictions. From these discrepancies we identify the key deficits of the current version of the model.

## INTRODUCTION

**Figure 1.**
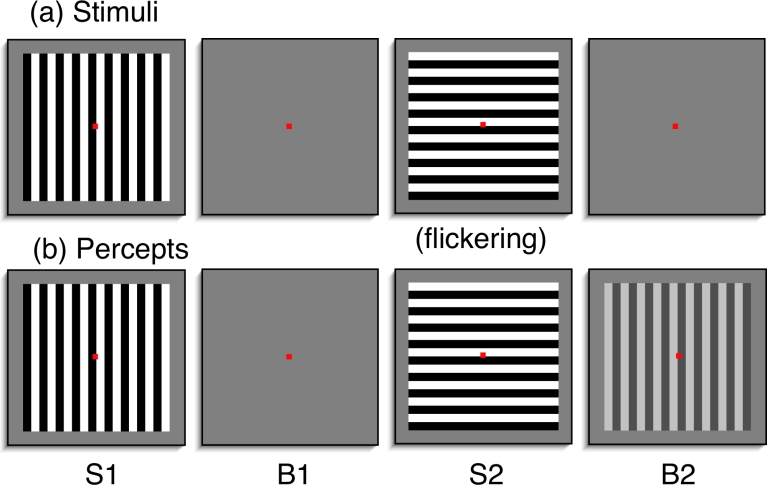
A schematic of the stimuli and percepts of the two-stimulus afterimage.

Francis and Rothmayer (2003) reported that sequential viewing of two orthogonally related patterns produces an afterimage percept related to the first pattern. They explained this afterimage using Grossberg’s (1994) FACADE theory. Figure 1a shows a sequence of images that produces the afterimage (Francis & Rothmayer, 2003). The first stimulus (S1) consisted of black and white vertical bars on a gray background that was presented for 1 s. S1 was replaced by a blank gray screen (B1) for a duration of 1 s. B1 was then replaced by a second stimulus (S2), which was made of horizontal black and white bars that flickered with their achromatic color complements. Finally, the observer was shown another blank screen (B2) and at the end of this blank the observer was asked to report on any afterimages. 

Figure 1b shows the percepts associated with the presentation of images. When observers were presented with a vertical or horizontal grating, observers veridically saw those images. During B1 observers did not see any afterimages, but during B2, observers reported seeing a vertical afterimage similar to S1. If S1 and S2 were of the same orientation, for example if both were horizontal gratings, observers reported few, if any afterimages.

These afterimages are probably the same type as the afterimages reported by Vidyasagar et al. (1999) . They showed a repeating sequence of radial arcs, blank screen, concentric circles, and a blank screen. Observers reported seeing an afterimage during the presentation of blank screens. Offset of the arcs produced an afterimage of concentric circles, while offset of the concentric circles produced an afterimage of radial arcs. 

**Figure 2.**
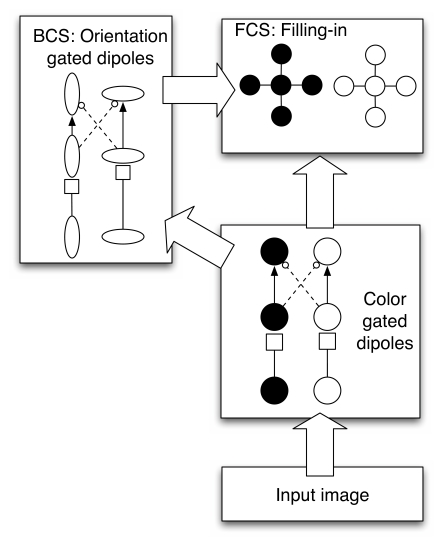
A schematic of the main components of FACADE theory. The input image feeds into a retinotopic representation of black and white, which compete in a gated dipole circuit. The gated dipole circuit produces complementary after-responses. The black and white information then feeds into edge detection in the BCS, which also contains a gated dipole circuit whose after-responses code orthogonal orientations. The edges in the BCS guide the spread of black and white information in the FCS filling-in stage to limit the spread of color and brightness information.

Francis and Rothmayer (2003) and Francis and Schoonveld (2005) reported simulations of Grossberg’s (1994) FACADE model that accounted for the appearance of the afterimage. In this theory, two separate pathways are used to compute visual information. Figure 2 shows a schematic of the major parts of the model. A boundary contour system (BCS) processes boundary or edge information, while a feature contour system (FCS) uses information from the BCS to allow diffusive filling-in of surface properties like color and brightness. The BCS detects oriented edges. The FCS uses the BCS information to determine where information spreads, leading to the final percept. 

Embedded within the FACADE architecture are gated dipole circuits (Grossberg, 1972). A gated dipole contains two pathways that compete as signals pass from lower to higher levels. A signal passing through one pathway inhibits a signal passing through the competing pathway. At offset of stimulation, a gated-dipole circuit produces a reduction in cross channel inhibition from the stimulated channel to the unstimulated channel. This reduction in inhibition leads to a rebound of activity in the unstimulated pathway. In the FACADE model, the properties of the gated dipole help to act as a reset signal to reduce persisting neural signals (Francis, Grossberg, & Mingolla, 1994).

**Figure 3.**
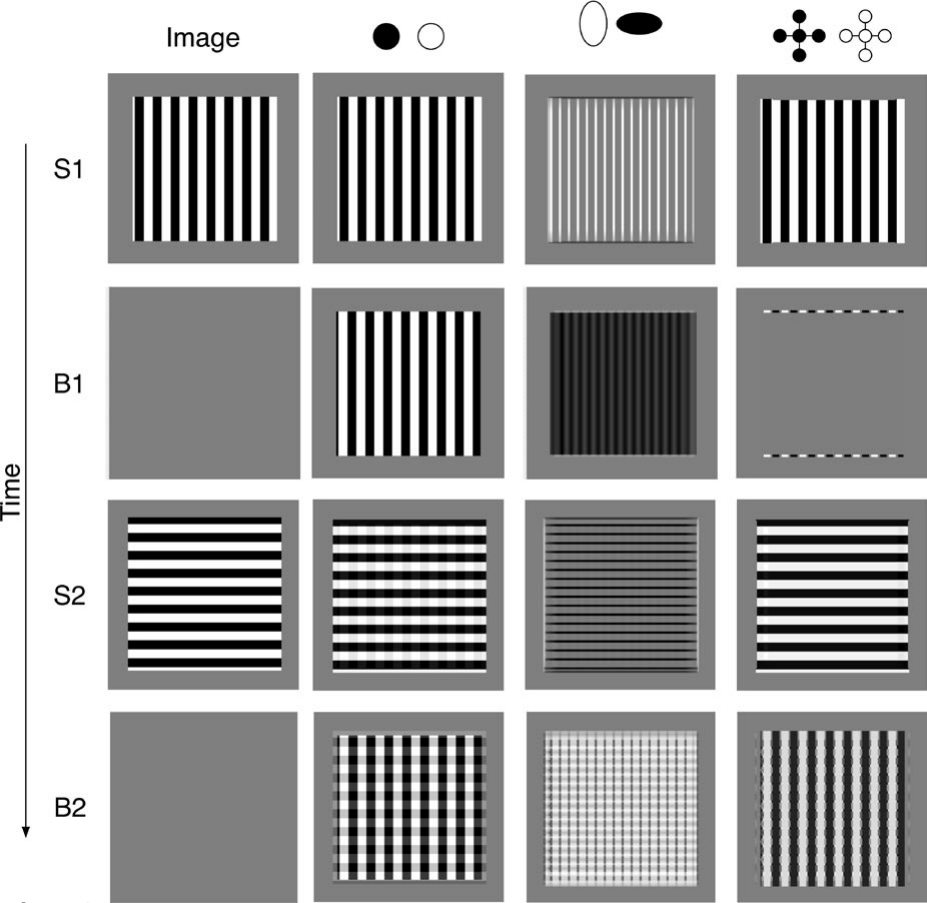
The results of a simulation of the model. During presentation of a vertical bar grating (S1), there are strong vertical boundaries and a vertical bar grating is present at the filling-instage.When the vertical bar grating is replaced by a blank screen(B1),there are color-complement after-responses and orientation after-responses. This combination of signals does not support an afterimage at the filling-instage.When the blank screen is replaced by a horizontal grating(S2),a veridical percept is again produced at the filling-instage. When the horizontal grating is replaced by another blank screen(B2),an after image orthogonal to S2 is produced at the filling-instage.

There are separate gated dipole circuits in the FACADE architecture that code for color and orientation. (In all of the discussions in this paper, we consider only achromatic colors.) Thus, at each pixel location there are two types of after-responses in the model. One codes the opposite color (black or white in the current simulations) and the other codes the opposite orientation (vertical or horizontal in the current simulations). The color after-responses are probably related to retinal afterimages (Kelly & Martinez-Uriegas, 1993), while Francis & Grossberg (1996) related the orientation afterimages to the complementary afterimages noted by MacKay (1957) . The combination of after-responses will produce a visible afterimage percept only if the oriented boundary signals separate the color signals into distinct regions at the filling-in stage. That afterimages involve a combination of retinal and cortical after-responses was suggested by Georgeson and Turner (1985) as a way of providing a qualitative explanation of afterimages of sine and square wave gratings. Suzuki and Grabowecky (2003) also suggested that afterimages may involve several different types of after-responses. Our work shows how this qualitative idea is part of a quantitative model whose mechanisms have previously been used to address entirely different data sets. The results of a simulation of the model with these interactions are shown in Figure 3, which shows the behavior of various stages of the model during a simulated two-stimulus afterimage trial. 

The trial starts with the presentation of S1, a vertical black and white grating. The output of the color gated dipole (indicated by the black and white circles) shows the input from the vertical grating. The boundary signals (marked by the oriented ovals) are primarily vertical. (Black color at a pixel indicates a response from a horizontally tuned cell, and white color at a pixel indicates a response from a vertically tuned cell.) The filling-in stage shows a vertical grating, and thus a veridical percept of S1. All of the simulation images in Figure 3 show the pattern of cell activities at the end of each stimulus duration. The color values in the image correspond to a difference in activity of the cells at that position (e.g., white minus black or vertical minus horizontal). The largest positive value is set equal to white, and the largest negative value is set equal to black. The value zero is always set to middle gray, and other positive and negative values are then scaled linearly to other gray values.

B1 is a blank that lasts for 1 s after offset of S1. Two kinds of after-responses are generated. At the color gated dipole, the active color at each pixel is flipped so that what was black is now white and vice-versa. Likewise, at the orientation gated dipole, what was once vertical is now horizontal and vice-versa. In addition, boundary grouping in the BCS completes across the gaps between the vertically arranged horizontal orientations. As a result, there is a mass of dense horizontal signals. When the vertically arranged color after-responses are joined with the horizontal orientation after-responses at the filling-in stage, no afterimage percept is produced. This is because the horizontal orientations allow color to flow left and right but not up and down. As a result, the black and white bars from the color signals spread over each other and cancel out. Except for a few (very weak) edge effects, there is no visible afterimage at the filling-in stage.

S2 consists of a horizontal grating. As in the experiments of Francis and Rothmayer (2003) , this horizontal grating flickered with its color complement, and what is shown in Figure 3 is the behavior of the model at the end of the last horizontal grating. The output of the color gated dipole shows predominately horizontally arranged black and white color signals, which are driven by the horizontal grating. However, faintly superimposed on the horizontal pattern are black and white vertical bars. (The faint vertical stripes may not be visible in the reproduction of the image.) These vertical stripes are color after-responses produced by the offset of the S1 vertical grating. The orientation signals are predominately horizontal (black) because the presentation of S2 produces strong responses among horizontally tuned cells at the appropriate positions on the edges of the bars. The faint vertical stripes are too weak to produce any vertical boundaries. The filling-in stage shows a horizontal grating, which corresponds to a veridical percept. 

B2 is a blank duration of 1 s after offset of S2. The responses of the color gated dipoles are a mix of black and white from S1 and S2. The orientation signals are primarily vertical, because offset of S2 produced after-responses among vertically tuned cells. The filling-in stage for B2 shows a vertical bar grating, which corresponds to the afterimage percept. The filling-in stage produces this pattern because the vertical boundary signals constrain the filling-in signals to spread only up and down, not left and right. Thus, the dark and light horizontal rows of inputs from the color gated dipoles spread across each other and cancel out. On the other hand, the dark and light columns across the color gated dipoles are kept separate and so support activity at the filling-in stage. The net effect is that the orientation after-responses force the filling-in stage to “pick out” the vertical pattern in the outputs of the color gated dipoles. In the model, the spatial structure of the perceived afterimage is a combination of the spatial layout of the color after-responses from S1 and the orientation after-responses from S2. If these two types of after-responses are not consistent with each other, then no afterimage should be created.

In the present study, we explored the effect of varying the relative orientation of the inducing stimuli. Previously (Francis & Rothmayer, 2003) we showed that in both the model and experimental data an MCAI percept appears when the inducing stimuli have orthogonal orientations, but not when they have parallel orientations. We now investigate the behavior of the model and experimental data to intermediate orientation differences.

## Experiment 1: Orientation tuning of the afterimage

### Model behavior

**Figure 4.**
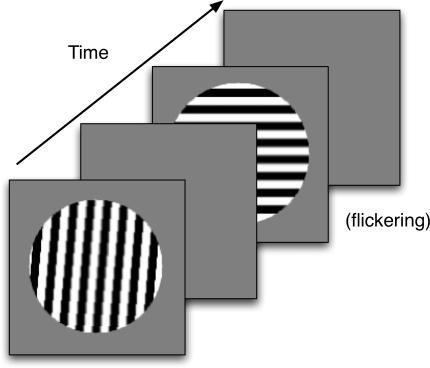
The sequence of images for a simulated trial in Experiment 1. The orientation of the first stimulus varied from trial to trial. Any after images were measured during the second blank frame.

All of the model simulations used the same equations and parameters as Wede and Francis (2006) . Figure 4 shows the sequence of stimuli presented to the model. S1 was a bar grating, placed within a circular aperture and presented for one simulated second. On different trials, S1 was rotated relative to S2. This was followed by a blank screen for 100 ms. S2 was always oriented horizontally and presented for a total of 2 s. To minimize color adaptation to S2, the bar grating flickered with an alternating phase shifted version of the grating (black and white bars changed to their opposite color). S2 was followed by a blank screen for 2 s, and at the end of the blank screen the model’s predicted percept was computed at the filling-in stage of the model.

**Figure 5.**
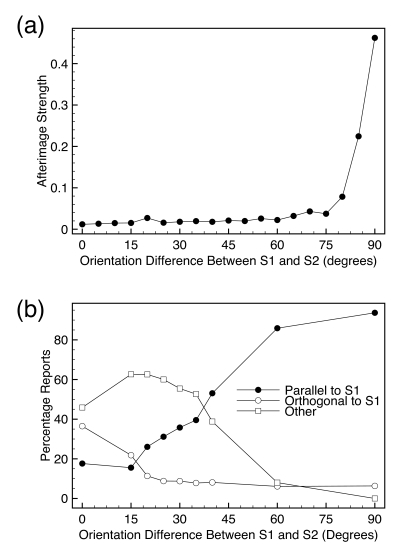
Results from Experiment 1. (a) The model predicts a strong afterimage when S1 and S2 differ by 90°. Small deviations from orthogonality lead to large decreases in the strength of the afterimage, (b) the experimental data show that reports of an afterimage parallel to S1 depend on the orientation difference between S1 and S2, but that the drop off in afterimage strength is not as rapid as predicted by the model. Any afterimages were measured during the second blank frame.

Figure 5a plots a measure of the strength of the afterimage in the model. Afterimage strength is calculated as the magnitude of the strongest black signal added to the magnitude of the strongest white signal across the filling-in stage of the model. Larger afterimage strength numbers indicate larger differences between those areas of the filling-in stage of the model for representing different values of gray. The model predicts that the relative orientations of S1 and S2 should have a large impact on the appearance of the afterimage. When the stimuli are orthogonal, the afterimage strength is at its strongest level. As the angle between the stimuli decreases the afterimage strength rapidly drops off, becoming half its peak value when S1 differs in only five degrees of rotation from the orthogonal of S2.

**Figure 6.**
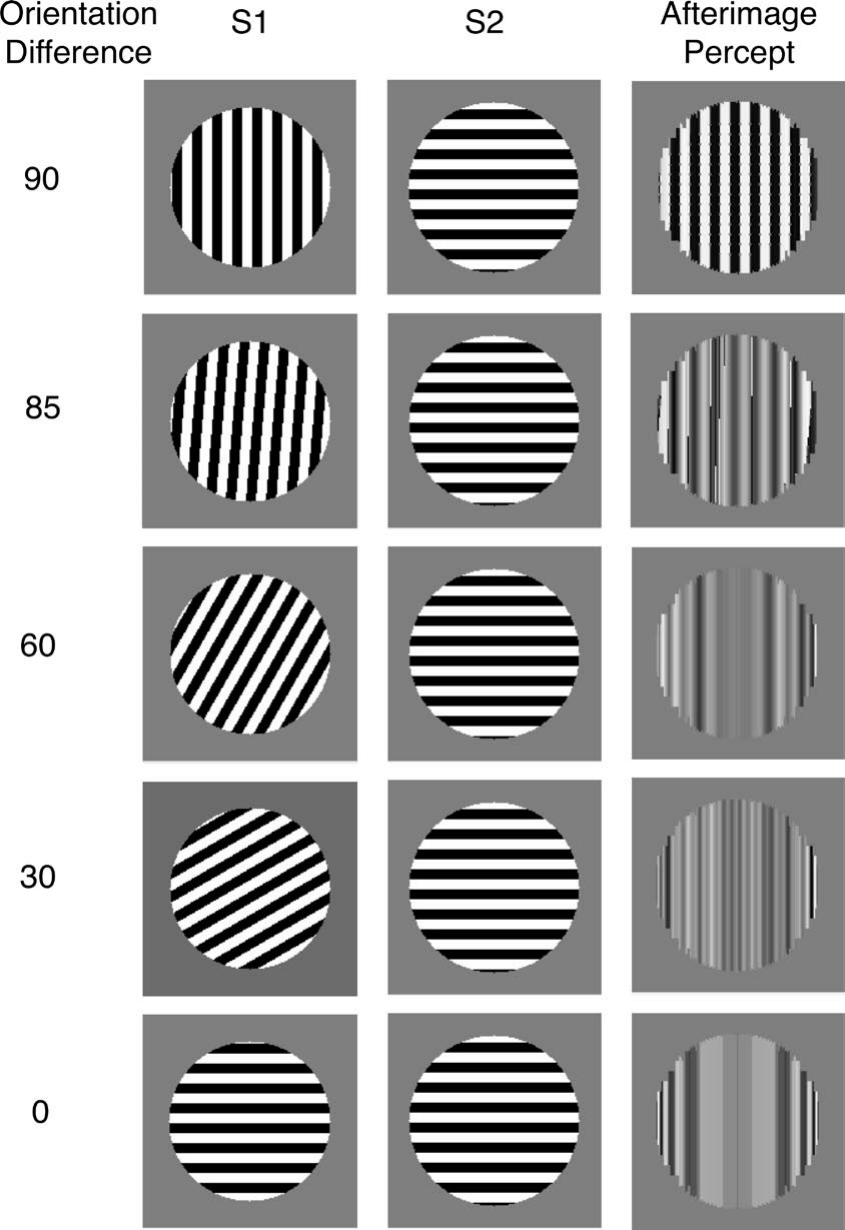
The spatial structure of the model-produced afterimage for various S1 and S2 orientation differences. The model always predicts that the afterimage is orthogonal to S2, regardless of the orientation of S1.

Figure 6 shows the spatial structure of the predicted afterimage percept for different combinations of S1 and S2 relative orientations. When S1 is orthogonal to S2, the afterimage percept consists of alternating vertical bars. When S1 is rotated only 5° clockwise from orthogonal, the afterimage percept is more muddled. This is because of the spatial interactions of the orientation after-responses from S2 and the color after-responses from S1. The orientation after-responses from S2 are all vertical and constrain whatever color after-responses exist to only flow up and down, not left or right. Because of the orientation of S1 and the thickness of the bars, there are differences in the proportion of black and white after-responses in different vertical columns. When there is more white than black in a column, the afterimage percept at that column will be light gray. Similarly, other columns will have an afterimage percept of dark gray, when there are more black than white after-responses. For further rotations (and smaller angle differences between S1 and S2) the number of black and white after-responses in a column tend to balance out with only small differences being present between different columns.

Because the activities across the filling-in stage are normalized in Figure 6, it is not meaningful to compare the strength of the signals across the different S1 orientations. As Figure 5a shows, when S1 and S2 are even slightly non-orthogonal, the afterimage signals are quite small. A key property though, is that regardless of the strength of the afterimage, the orientation of bars in the afterimage are vertical, that is, orthogonal to the orientation of S2. This is inherent in the structure of the model. The afterimage percept is constructed by the flow of color after-responses from S1 being constrained by the direction of orientation after-responses from S2. According to the model, the flow must always be orthogonal to the orientation of S2.

So the model makes two main predictions. First, the visibility of the afterimage (measured as the difference between visible black and white signals in the percept) should rapidly decrease as the relative orientations of S1 and S2 deviate from perpendicular. Second, the shape of the perceived afterimage should always be orthogonal to the orientation of S2.

### Method and procedure

Twenty-one students from the Purdue University subject pool participated in the experiment in return for course credit. Each observer reported normal or corrected-to-normal vision. Observers were shown all stimuli in a lit room on a computer monitor that was operating at 75 Hz. The stimuli were created and run with MATLAB and the Psychophysics Toolbox extensions package (Brainard, 1997; Pelli, 1997), on a Windows XP operating system. Observers started each trial with a key press, which was followed by the presentation of the stimuli, all of which were shown with a gray background and viewed at a distance of 39 cm.

The stimuli were generally the same as for the model simulations, with one notable exception. For technical reasons, having to do with undersampling of orientations, the simulations are best run with variations in the orientation of S1, relative to a fixed S2. This insures that the orientation after-responses generated by S2 are constant from one condition to the other. In contrast, pilot experimental work indicated that it would be easier for observers to make consistent responses if the orientation of S1 was held fixed and the orientation of S2 was rotated across conditions. If the model mechanisms are valid, the variable that matters is the relative orientation of the stimuli, so the two situations should be equivalent.

**Figure 7.**
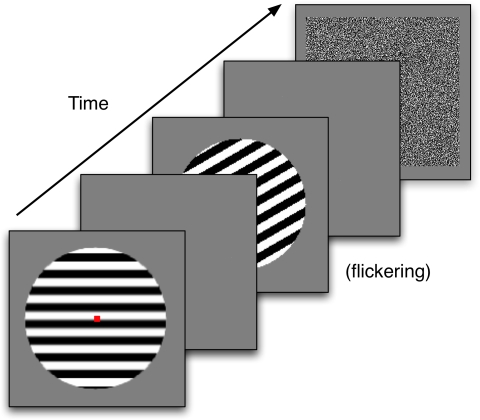
The sequence of images for an experimental trial in Experiment 1. The orientation of the second stimulus varied from trial to trial. Any afterimages were measured during the second blank frame.

Figure 7 schematizes a trial with S1 as a horizontal bar grating. On half the trials S1 was a vertical grating. The grating was presented within a circular aperture, which had a diameter of 14.6° of visual angle. The grating consisted of 16 equally-sized bars that alternated in color between black and white. There was also a small red box in the middle of the display, which was to be used as a fixation point. S1 was shown for 1 s. A blank gray screen with a fixation point followed the first stimulus for 100 ms. Gray, white, and black had a luminance of 49,100 and 1.3 cd/m2, respectively. Each luminance measurement was taken from a patch of color that filled the aperture of a light meter.

S2 consisted of a bar grating that was rotated counter clockwise at various angles from that of the first stimulus. The angles of rotation used for Experiment 1 were 0, 15, 20, 25, 30, 35, 40, 60, and 90°. The rotated bar gratings consisted of 16 bars that alternated in color between black and white. The second stimulus flickered back and forth between two bar gratings of the same orientation but with colors of opposite polarity. Each flicker frame was displayed for 100 ms and each of the color complementary frames was shown 10 times for a total exposure to S2 of 2 s.

Following S2, observers were shown a blank gray screen with a fixation point, for one second, followed by a field of random dots that covered the area where the previously presented stimuli had been shown. Observers were then prompted to identify what they saw in the blank gray screen, just before the dots appeared. Observers were able to respond in one of three ways with a corresponding key press to indicate that they saw a vertical afterimage, a horizontal afterimage, or other. A vertical afterimage response indicated the observer saw vertically oriented light and dark bars. A horizontal afterimage response indicated the observer saw horizontally oriented light and dark bars. The other response indicated that the observer saw no afterimage or saw an afterimage but it was something other than vertical or horizontal bars.

All possible combinations of S1 (two orientations) and S2 (nine orientations) were replicated twice for a total of 36 trials. There was a 12 s delay between trials to minimize any carryover effects from one trial to the next.

## Results

Figure 5b plots the percentage of afterimage reports as a function of the orientation difference between S1 and S2. There were no notable differences between conditions when S1 was vertical or horizontal, so the data were combined across these conditions. Observers reported an afterimage parallel to S1 most often when S2 was orthogonal to S1. As the orientation of S2 shifted to being parallel to S1, observers increased reports of other. When S2 was parallel to S1 there was a slightly increased tendency to report an afterimage orthogonal to S1. This was probably a complementary orientation afterimage (MacKay, 1957), which looks rather different from the other afterimages reported here.

Consistent with the previous findings of Francis and Rothmayer (2003) and the model simulations, an afterimage parallel to S1 was most common when S2 was orthogonal to S1 and least likely when S2 was parallel to S1. However, the new findings differ dramatically from the model simulations when S2 takes an intermediate orientation relative to S1. While the model predicts that afterimage appearance weakens quite sharply as S2 differs from being orthogonal to S1, the experimental data shows a gradual change in reports of an afterimage parallel to S1. When S2 was rotated 60° from S1, observers reported seeing an afterimage parallel to S1 over 80% of the time. Even when there was only a 30° difference between S1 and S2, observers reported an afterimage almost 35% of the time. 

At the end of the experiment, we asked observers whether they saw afterimages that were not horizontal or vertical. The model predicted that the perceived afterimage should be orthogonal to S2, but all observers reported that the perceived afterimage was related to the shape of S1 rather than to S2.

### Discussion

The results of Experiment 1 are contrary to the model’s quantitative predictions. While the model predicted a rapid decrease in afterimage visibility as relative orientation between S1 and S2 differed from orthogonal, the data found a quite gradual decrease. This discrepancy is significant because the prediction was based on a fundamental aspect of how the model accounts for the creation of the afterimage percept.

We should note that our critique of the model’s behavior only makes sense if we believe that the model’s reported strength of the afterimage can be meaningfully compared to the percentage reports of the afterimage among our observers. Previous research has found a strong correlation between the model strength and percentage reports. In a study of various inducer durations, Wede and Francis (2006) reported that model strength and percentage reports had a correlation of *r* = .92. Likewise, in a study of attention effects on these types of afterimages (Wede & Francis, in press), we found that model strength and percentage reports had a correlation of *r* = .97. In contrast, the correlation between the predicted and experimentally observed data in Experiment 1 is only *r* = .69, and there are notable differences in the data curves. 

The discrepancy between the predicted and observed results cannot be accommodated with a simple change in model parameters. For the model to explain the absence of an afterimage percept when S1 and S2 are parallel, it must allow color to spread in such a way that the dark and light filling-in regions cancel each other out. At the same time, dark and light filling-in regions must remain separated when S1 and S2 are orthogonal, else their signals will cancel and no afterimage will be generated. These two constraints are met by allowing color signals to flow in the direction of an oriented boundary but not in the orthogonal orientation. But this solution necessarily leads to the conclusion that the dark and light filling-in regions must cancel out when S2 is slightly off orthogonal. Since the data do not match this prediction, it appears that there is a fundamental problem with the model’s explanation of these afterimages.

Less quantitative but equally important were the observers’ reports that the afterimage shape was related to the shape of S1 rather than S2. The observer reports agreed with our own phenomenological experience of the afterimage shape. Again, the model prediction of the afterimage shape is a necessary property of its current explanation of these afterimages and it does not appear that any change of parameters will lead to fundamentally different model behavior. We explored this issue further in Experiment 2.

## Experiment 2: Orientation tuning with a grid inducer

 The results from Experiment 1 were surprising because they challenged some of the basic mechanisms of the model; mechanisms that had correctly predicted data about these kinds of afterimages. Francis and Schoonveld (2005) analyzed the model and noted that it predicted that the shape of the afterimage was a joint construction of after-responses from S1 and S2. To test this idea, they used a grid for S1 and an oriented grating for S2. The model predicted, and experimental data verified, that the perceived afterimage shape was orthogonal to the orientation of S2, which picked out only one orientation from S1. However, Francis and Schoonveld (2005) used only vertical and horizontal elements for their stimuli. We now further analyze the model’s behavior for similar inducing stimuli, but with more orientation differences. 

### Model behavior

**Figure 8.**
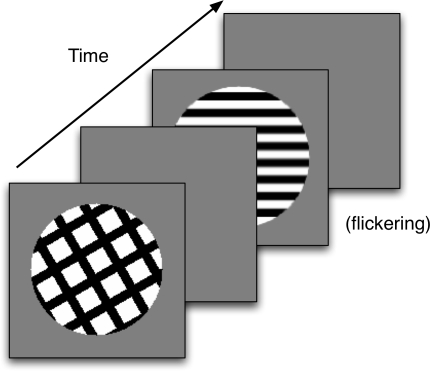
The sequence of images for a simulated trial in Experiment 2. The orientation of the first stimulus varied from trial to trial. Any afterimages were measured during the second blank frame.

Figure 8 shows the sequence of stimuli presented to the model. S1 was a hatched pattern of five black bars on a white circular background that was presented for one simulated second. On different trials, S1 was rotated to different orientations. S1 was followed by a blank screen for 100 ms. S2 was always oriented horizontally and presented for a total of 2 s. To minimize color adaptation to S2, the bar grating flickered with an alternating phase shifted version of the grating (black and white bars changed to their opposite color). S2 was followed by a blank screen for 2 s, and at the end of the blank screen, the model’s predicted percept was computed at the filling-in stage of the model.

**Figure 9.**
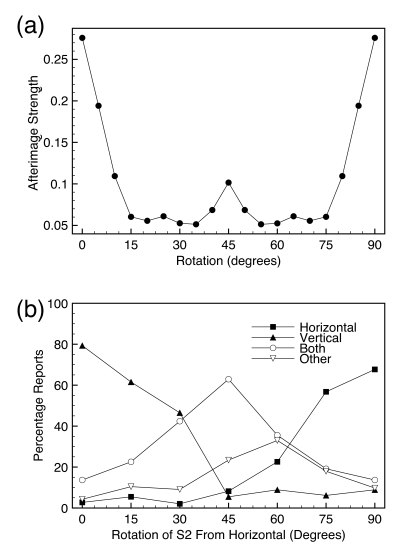
Results from Experiment 2. (a) The model predicts a strong afterimage when S2 differs by 90 degrees from either of the components of S1. Small deviations from orthogonality lead to large decreases in the strength of the afterimage, (b) the experimental data show that reports of horizontal and vertical afterimages occur for rotations where the model predicts weak afterimages; moreover, the data show that observers report seeing both horizontal and vertical components of the afterimage for intermediate rotations.

Figure 9a plots a measure of the strength of the afterimage in the model as a function of the rotation of S1. This strength calculation does not consider the shape of the afterimage percept, but as shown below the model makes a straightforward prediction regarding the after-image shape. The results for rotations up to 45° are similar as those in Figure 5a. There is a rapid drop in afterimage strength. There is a slight upturn in afterimage strength at 45° and then a symmetrical increase in afterimage strength for larger rotations. The symmetry occurs because the pattern of S1 repeats after a 45° rotation. The upturn at 45° occurs because when the pattern is at 45° the intersections of the crossed bars line up vertically.

**Figure 10.**
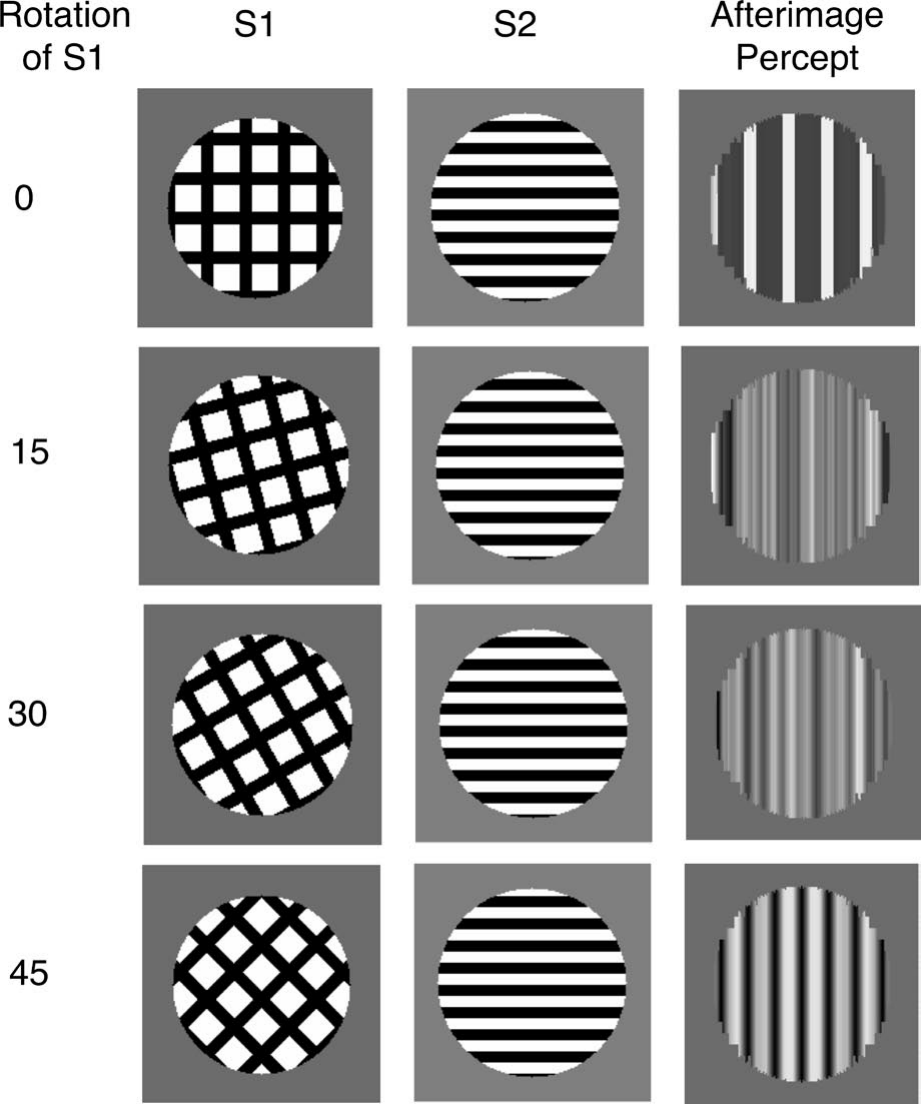
The spatial structure of the model-produced afterimage for various S1 and S2 orientation differences. The model always predicts that the afterimage is orthogonal to S2, regardless of the orientation of S1.

The more significant behavior of the model is the predicted shape of the afterimage, which is shown in Figure 10. As in Experiment 1, the model predicts that the shape of the afterimage should always be of a bar grating orthogonal to the orientation of S2. For these simulations S2 was always horizontal, so the orientation of the afterimage was always vertical. Notice that for no orientation does the model ever predict that both vertical and horizontal components of S1 will be part of the afterimage. Indeed, the orientation after-responses from S2, which guide the filling-in of color signals, cannot support the simultaneous presence of both vertical and horizontal components of S1. An analogous pattern of results could be created for any orientation of S2, with the perceived afterimage always being a bar grating that is orthogonal to the S2 orientation.

The model predictions are variations of those in Experiment 1. However, the design of the experiment allows for a more precise measurement of the afterimage shape from observers. The experimental method was quite similar to that used in Experiment 1, but a few changes were made to be more similar to the methods used by Francis and Schoonveld (2005) , to make the task easier for observers, and to work with different computer equipment.

### Method and procedure

Twenty-two students from the Purdue University subject pool participated in the second experiment in return for course credit. Each observer reported normal or corrected-to-normal vision. Observers were shown stimuli in a lit room on a PC with a Windows XP operating system and a computer monitor running at 85 Hz. Stimuli were created and shown with MATLAB and utilized the Psychophysics Toolbox extension (Brainard, 1997; Pelli, 1997).

**Figure 11.**
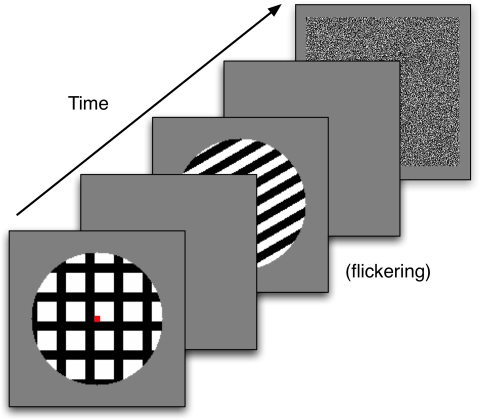
The sequence of images for an experimental trial in Experiment 2. The orientation of the second stimulus varied from trial to trial. Any afterimages were measured during the second blank frame.

Figure 11 provides a schematic for one of the trials a observer might observe during the experiment. Observers started each trial with a key press, which was followed by the presentation of stimuli, which were shown on a gray background and viewed at a distance of 45 cm. S1 consisted of two intersecting bar gratings. The gratings were presented within a circular aperture that had a diameter of 10 cm (12.6° of visual angle) and were shown for 1 s. Each grating consisted of five vertical black bars that intersected with five horizontal black bars on a white background. A small red box was placed in the middle of the display as a fixation point. Gray, white, and black had a luminance of 40, 97, and 0.5 cd/m2, respectively.

S1 was followed by a blank gray screen, which included the fixation point and was shown for 106 ms, and was immediately followed by S2, which consisted of either a bar grating or a blank gray screen. The possible bar grating orientations were 0, 15, 30, 45, 60, 75, and 90° from horizontal. The bar grating consisted of 16 bars that alternated in color between black and white. S2 flickered, as it did in Experiment 1, back and forth between two bar gratings of the same orientation, but with colors of opposite polarity. Each frame was displayed for 106 ms and each of the color complementary frames was shown 10 times for a total exposure to S2 of 2.12 s.

Immediately following S2, observers were shown a blank gray screen with a green fixation point for a time period of 1 s. A green fixation point was used rather than a red fixation point to aid observers in differentiating between the S2 blank and the subsequent blank where a response was to be determined. After the blank, observers were shown a field of random dots that covered the area where the prior stimuli had been shown and were prompted to identify what they saw in the prior screen when the fixation point turned green.

Observers were able to respond in one of four ways, with a corresponding key press. Response keys corresponded to a vertical afterimage, a horizontal afterimage, a both afterimage, or other. The vertical afterimage was described as vertically oriented light or dark bars, a horizontal afterimage was described as horizontally oriented light or dark bars, a both afterimage was described as both vertical and horizontal bars forming a grid type pattern, and the other response was pressed for trials in which no afterimage was perceived or if an afterimage was perceived, but it was something other than a vertical, horizontal, or both afterimage. There were eight possible second stimulus conditions and they were shown in a randomly displayed order. All conditions were replicated four times so each observer completed 32 trials. There was a 12 s delay between trials to minimize any carryover effects from one trial to the next.

### Results

Figure 9b plots the percentage of reports of various types of afterimages as a function of S2’s orientation relative to horizontal. Observers reported horizontal afterimages most often when S2 was oriented vertically and rarely when S2 was oriented horizontally. Observers reported vertical afterimages most often when S2 had a horizontal orientation, and these reports were drastically reduced when S2 had a vertical orientation. Reports of both horizontal and vertical bars were at a minimum for the extreme angles of S2. Reports of afterimages when S2 was blank were rare, with 75% of the responses being “other”.

 All of these results are a replication of the findings in Francis and Schoonveld (2005) . What is new is the reports for other S2 orientations. When S2 was at 45°, observers primarily reported seeing a both afterimage. Reports of a both afterimage fall off symmetrically for angles off of 45°, generally in favor of one orientation or the other. 

### Discussion

As in Experiment 1, the experimental data contradicts the basic principles of the model’s explanation for the afterimages. Figure 9 shows that the model predicts a much faster fall-off of afterimage strength as a function of S1 and S2 orientation differences than the data actually demonstrates. More significantly, though, the data in Figure 9b clearly show that observers saw both horizontal and vertical components in the afterimage for a broad band of S2 orientations. This report violates the model’s hypothesis that the afterimage is constructed by S1 color after-responses flowing along the S2 orientation after-responses. If that hypothesis held, the afterimage would appear to be orthogonal to S2 or not be visible at all, as shown in Figure 10.

## Conclusions

We have explored how orientation differences between S1 and S2 affected the shape and strength of the resulting afterimage. We analyzed the FACADE model for this situation and identified critical predictions. The experimental data to test those predictions do not support the model.

The result was surprising because the model has generally had great success at explaining and predicting the properties of these kinds of afterimages. Francis and Rothmayer (2003) showed how the model produces the afterimage percept and tested the model’s prediction that spatial frequency should have little effect on afterimage visibility while relative orientation (orthogonal or parallel) of S1 and S2 should have a big effect. Francis and Schoonveld (2005) predicted the shape of the afterimage when S1 was a hatched pattern. Wede and Francis (2006) analyzed the dynamics of the model after-responses and predicted afterimage strength as a function of relative delays between S1 and S2. Finally, Wede and Francis (in press) used the model to explain attention effects on this kind of afterimage and on negative afterimages. In all of these cases, the experimental data matched the model predictions quite well. 

However, there have been a few failures of the model, and those failures point toward a common problem in the current versions of the model. Francis and Ericson (2004) noted that if S1 had a blank gap separating left and right sides of horizontal bars, then the filling-in stage of the model should be able to fill-in that gap as color after-responses flow along the boundaries generated by offset of S2. Contrary to the model predictions, experimental data found that observers did see the gap. Similarly, Francis and Schoonveld (2005) noted that the model predicted that when the left and right sides of an S1 horizontal grating flipped polarity in the center, then the flow of color after-responses should cancel each other out and no afterimage should be seen. Once again, observers reported seeing an afterimage with sides of different polarity. 

The current findings seem to be of the same sort. The model predicts that color signals should cancel out when they flow along the boundaries generated by the offset of S2. However, the experimental data suggests that the color signals do not cancel out as readily as the model predicts. The canceling of color signals is an integral part of the model’s behavior because it explains why no afterimage is seen when S1 and S2 are parallel bar gratings and why a hatched grating for S1 can produce an afterimage of only a bar grating (Francis & Schoonveld, 2005).

Thus, all of the model failures appear to be related to properties of the filling-in stage of the model. Traditionally, the color signals at the filling-in stage behave like a passive diffusion process (Gerrits & Vendrick, 1970; Grossberg & Todorovic, 1988; Paradiso & Nakayama, 1991; Grossberg & Hong, 2006). This appears to be an inaccurate description of the filling-in stage. Regrettably, proposed alternative mechanisms (Francis & Ericson, 2004) do not address the current problems with the model.

Further modeling work is needed to identify a filling-in mechanism that can account for the properties of these afterimages and remain consistent with the other uses of filling-in. The properties of two-stimulus afterimages appear to be a useful tool for exploring filling-in mechanisms and further study of these afterimages may help identify alternative filling-in mechanisms.
